# A new stem group echinoid from the Triassic of China leads to a revised macroevolutionary history of echinoids during the end-Permian mass extinction

**DOI:** 10.1098/rsos.171548

**Published:** 2018-01-31

**Authors:** Jeffrey R. Thompson, Shi-xue Hu, Qi-Yue Zhang, Elizabeth Petsios, Laura J. Cotton, Jin-Yuan Huang, Chang-yong Zhou, Wen Wen, David J. Bottjer

**Affiliations:** 1 Department of Earth Sciences, University of Southern California, Zumberge Hall of Science, 3651 Trousdale Pkwy, Los Angeles, CA 90089-0740, USA; 2 Chengdu Institute of Geology and Mineral Resources, Chengdu 610081, People's Republic of China; 3 Chengdu Center of China Geological Survey, Chengdu 610081, People's Republic of China; 4 Florida Museum of Natural History, University of Florida, 1659 Museum Road, PO Box 117800, Gainesville, FL 32611, USA; 5 Department of Geological Sciences, University of Florida, 241 Williamson Hall, PO Box 112120, Gainesville, FL 32611-2120, USA

**Keywords:** sea urchin, Triassic, Lazarus effect, echinoderm, Luoping Biota

## Abstract

The Permian–Triassic bottleneck has long been thought to have drastically altered the course of echinoid evolution, with the extinction of the entire echinoid stem group having taken place during the end-Permian mass extinction. The Early Triassic fossil record of echinoids is, however, sparse, and new fossils are paving the way for a revised interpretation of the evolutionary history of echinoids during the Permian–Triassic crisis and Early Mesozoic. A new species of echinoid, *Yunnanechinus luopingensis* n. sp. recovered from the Middle Triassic (Anisian) Luoping Biota fossil Lagerstätte of South China, displays morphologies that are not characteristic of the echinoid crown group. We have used phylogenetic analyses to further demonstrate that *Yunnanechinus* is not a member of the echinoid crown group. Thus a clade of stem group echinoids survived into the Middle Triassic, enduring the global crisis that characterized the end-Permian and Early Triassic. Therefore, stem group echinoids did not go extinct during the Palaeozoic, as previously thought, and appear to have coexisted with the echinoid crown group for at least 23 million years. Stem group echinoids thus exhibited the Lazarus effect during the latest Permian and Early Triassic, while crown group echinoids did not.

## 1. Introduction

The effect of the end-Permian mass extinction on the macroevolutionary history of echinoids has become a classic example of the extinction event's devastating influence on the macroevolutionary history of metazoans [1–4]. The long-accepted model proposed that only a single lineage, that of the genus *Eotiaris* (previously called *Miocidaris*), belonging to the crown group echinoid family Miocidaridae was the only lineage of echinoid to survive the extinction event, and all post-Palaeozoic echinoids could, thus, trace their origin back to *Eotiaris* [2,5,6]. New fossil finds [7] are beginning to shift this paradigm, as echinoid taxa once thought exclusive to the Palaeozoic have been found in Triassic strata. Palaeozoic echinoids, which make up the majority of the clade's stem group, have tests composed of multiple columns of interambulacral and ambulacral plates which articulate flexibly and disarticulated rapidly following death [8,9]. This is in stark contrast to the echinoid crown group, which has a test structure consisting of only two columns of ambulacral plates, and two columns of interambulacral plates, and in many taxa displays test plating with interlocking stereom [6,10]. Further phylogenetic analyses and analyses of ghost lineages have shown that, although the miocidarids were the only echinoids with fossil representation in both the Palaeozoic and the Mesozoic, one or two other lineages of crown group echinoids probably also crossed the Permian–Triassic boundary [11–13]. Nevertheless, it was accepted that the Permian–Triassic extinction spelled the end for the echinoid stem group, which presumably never survived into the Mesozoic.

**Figure 1.**
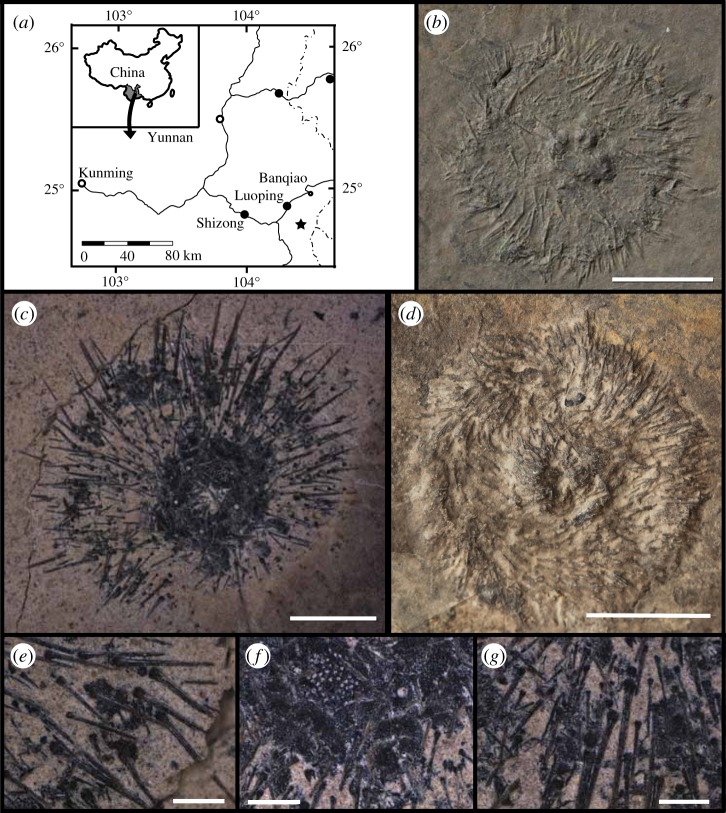
Specimens and location of *Y. luopingensis* n. sp. (*a*) Locality map showing the location of the Luoping Biota marked as star. Adapted from [18] (*b*) specimen 61701; note the bulge in the centre of the test which probably indicates the Aristotle's lantern inside of the compressed test. (*c*) Specimen 32321 which shows an apical view of a compressed test with apical disc with genital plates, an ocular plate and the madreporite. (*d*) Specimen 61163 showing a compressed test with spines. (*e*) Close-up of spines and ambulacral plate on specimen 32321. Note the absence of a milled ring and the striate nature of the spines. (*f*) Close-up view of the madreporite, ocular plate and adapical coronal plating of specimen 32321. Note the imbrication of the plates, with more adoral plating imbricating over more adapical plates. (*g*) Close-up of coronal plating and spines on specimen 32321. Spines and tubercles are arranged in distinct rows with larger spines lying slightly below corresponding imperforate and non-crenulate tubercles. Scale bars in (*b*,*d*) are 1 cm, bar in (*c*) is 2 mm and bars in (*e–g*) are 500 µm.

The fossil record of echinoids in the Early Triassic is, however, notoriously poor [12], and only three published localities have produced articulated specimens globally [5,14–16]. Stem group echinoids putatively assigned to the family Proterocidaridae have also recently been recovered from Middle and Upper Triassic strata, though not without controversy [17], and indicate that stem group echinoids did, in fact survive the Late Permian mass extinction. Additional fieldwork in the Middle Triassic of Yunnan Province, southwestern China (figure 1*a*), further indicates that this poor fossil record may have obscured the true macroevolutionary history of echinoids throughout the Early Triassic. New fossil specimens from the Anisian Luoping Biota [18] indicate that stem group echinoids were widely distributed in the Triassic. Phylogenetic analyses of Palaeozoic and Early Mesozoic echinoids, including this new species *Yunnanechinus luopingensis* n. sp. (figure 1*b–g*), indicate that it is a stem group echinoid and re-affirm that multiple lineages of echinoids crossed the Permian–Triassic boundary. Thus, multiple Palaeozoic echinoid clades survived the biotic and environmental turmoil characterizing the latest Permian and Early Triassic.

## 2. Material and methods

In order to determine whether or not *Yunnanechinus* represented a stem group echinoid which survived the end-Permian mass extinction event, we used parsimony-based and Bayesian phylogenetic analyses to determine its phylogenetic placement with respect to 13 stem and crown group echinoids spanning the Tournaisian (Early Mississippian) to Rhaetian (Late Triassic). The Silurian echinoid *Echinocystites ponum* was used as an outgroup. Our character matrix consisted of 69 characters coded for 14 taxa. Fourteen characters were multistate, while 55 were binary. All but one character were unordered. Parsimony analyses were run in PAUP* [19] using a heuristic search with 1000 random addition sequence replicates using starting trees obtained using stepwise addition and branch swapping through tree bisection and reconnection. Our Bayesian analyses were run in MrBayes v. 3.2 [20] using the Mk model of character change [21], which, in a Bayesian framework, has recently been demonstrated to estimate phylogenetic relationships more accurately than parsimony-based methods [22,23]. In order to account for ascertainment bias, as there were no constant characters included in the dataset, character coding was set to variable. We used a gamma distribution with a prior of exponential (1.0) to model rate variation and the prior on unconstrained branch lengths was also exponential (1.0). We used a symmetric Dirichlet prior on character state frequencies with parameter *α *= ∞. The joint posterior distribution of trees, branch lengths, and parameters was estimated using Markov chain Monte Carlo (MCMC). Details of MCMC are shown in electronic supplementary material. We additionally used sensitivity analyses to determine the robustness of our results to outgroup choice and model parameters. Further details of character scoring and sensitivity analyses can be found in electronic supplementary material. The institutional abbreviation LPI is the Invertebrate Palaeontology Collection, Chengdu Institute of Geology and Mineral Resources, Chengdu, China.

## 3. Systematic palaeontology

Echinoidea Leske, 1778

Stem group Echinoidea
Incertae familiae
Genus *Yunnanechinus* n. gen.
*Etymology*. Named for Yunnan, China from whence the type species is known.
*Diagnosis.* As for species
*Type species*. *Yunnanechinus luopingensis* n. sp.
*Occurrence*. As for species.
*Yunnanechinus luopingensis* n. sp.
urn:lsid:zoobank.org:act:19902A64-E79D-4E2C-ADB0-329CD565F2CB
*Etymology*. Named for the Luoping Biota, the fossil Lagerstätte from which the species is described.

*Diagnosis.* Test with imbricate plating, at least adapically and ambitally. Genital plates with one gonopore per plate (figure 1*f*). Plates of apical system covered with small, imperforate non-crenulate tubercles. Interambulacral plates polygonal to subpentagonal in shape. Interambulacral plates with a single imperforate non-crenulate tubercle, and sparse imperforate non-crenulate secondary tubercles. Spines less than half the diameter of the test in length, finely striate and without a milled ring (figure 1*e,g*).

*Material*. The holotype is specimen LPI-32321, paratypes are specimens LPI-2638, LPI-61163, and LPI-61701A,B.

*Occurrence*. All specimens from the Middle Anisian (Pelsonian) Guanling Formation of the Luoping Biota of Yunnan Province, South China.

*Description.* Test small to very small. Specimen 32321 6.48 mm in diameter, specimen 32220 15.99 mm in diameter, specimen 32638 is 13.31 mm in diameter, specimen LPI-61163 is 22.44 mm in diameter, specimen LPI-61701B is 21.31 mm in diameter. Test plating is imbricate, at least above the ambitus.

Peristomial plating and lantern unknown, but all specimens show a small bump located in the centre of the test, which probably represents the lantern inside the collapsed test. The apical disc is well preserved on specimen 32321 (electronic supplementary material, figure S4A, S4B). The madreporite is clearly visible atop interambulacrum number 2, while gonopores are visible on other genital plates. Each visible genital plate bears a single large gonopore. These gonopores are centrally located, except above interambulacrum 2, where the gonopore appears to be located adambulacrally to the madreporite (electronic supplementary material, figure S4A, S4B). At least one ocular plate appears to be present and appears to be in contact with the periproctal plates, indicating that at least some of the ocular plates are insert. These plates appear to be covered with numerous small secondary, imperforate and non-crenulate tubercles, which appear to have borne small, striate spines, morphologically similar to those on the rest of the test.

The interambulacral plating is largely obscured throughout the tests of all specimens except for specimen 32321, which displays interambulacral plating adapically and in a few places at and slightly above the ambitus (electronic supplementary material, figure S4A, S4B). The number of interambulacral plates in each interambulacral area is thus unknown. The shape of the plates is obscured near the ambitus, though adapically the plates are polygonal to subpentagonal in shape (electronic supplementary material, figure S4C). These plates imbricate adapically, with more adoral plates laying overtop of those more adapical plates. None of the plates appear to show any scrobicular ring or well-defined scrobicule. A single non-crenulate and imperforate primary tubercle is located subcentrally on the more adambital plates, while smaller primary tubercles are present on the more adapical plates (electronic supplementary material, figure S4C). In a few places on the test, these larger tubercles are associated with just barely disarticulated spines, indicating that there does appear to be size differentiation among tubercles and spines located more adambitally. On one plate, the presence of a smaller, imperforate and non-crenulate tubercle associated with a small spine confirms that these plates bear smaller secondary tubercles, though the exact number is unknown. A single ambulacral plate is present on specimen 32321 (figure 1*e*). The pore pair is oriented obliquely on the plate, though it is not possible to tell if the pore pair is angled adambulacrally or perradially. A small interporal partition separates the two pores of the plate. Above the pore pair is a single small imperforate and non-crenulate tubercle.

Small spines cover the tests of all specimens (electronic supplementary material, figure S4D–S4F) and, with the exception of specimen 32321, are all that is visible on the specimens. These spines are less than half the diameter of the test and taper distally. There are both primary and secondary spines, associated with primary and secondary tubercles, respectively, and both are identical in morphology except for their size. There is no milled ring and the spines bear fine striations. Proximally, all spines end in a small swelling, which is the base. The acetabulum is non-crenulate.

## 4. Results

**Figure 2.**
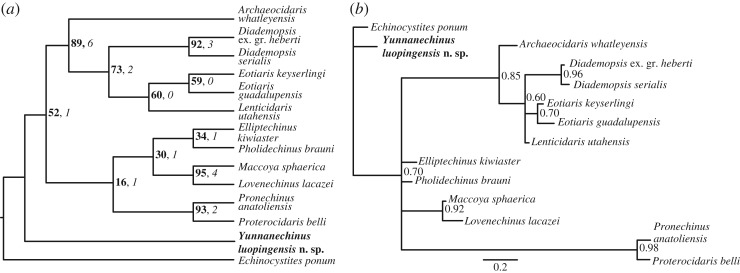
Results of phylogenetic analyses showing phylogenetic position of *Y. luopingensis* n. sp. (indicated in bold) relative to Late Palaeozoic and Early Mesozoic stem and crown group echinoid genera. (*a*) Single MPT resulting from equally weighted parsimony analyses using 69 characters and rooted on *Echinocystites ponum*. Tree length = 121 steps, CI = 0.69, RI = 0.74. Numbers at nodes in bold represent bootstrap proportions resulting from 10 000 ‘fast’ bootstrap replicates while italicized numbers are decay indices for each node. (*b*) Fifty percent majority rule consensus tree summarizing posterior distribution of trees resulting from Bayesian analyses using a symmetric Dirichlet prior with parameter *α* = ∞. Numbers at nodes represent PP of each node. Scale bar denotes scale for branch lengths, which are the average branch lengths from the posterior distribution of trees.

Bayesian and parsimony-based phylogenetic analyses are shown in figure 2, while results of sensitivity analyses are in electronic supplementary material, figures S1–S3. Parsimony analyses resolved a single most parsimonious tree (MPT) with length 121 steps, consistency index (CI) = 0.69, and retention index (RI) = 0.74. *Yunnanechinus* plotted as the most basal taxon in the ingroup, while two clades were more derived, one composed of proterocidarid, palaechinid and lepidocentrid echinoids, and one composed of *Archaeocidaris* and the echinoid crown group (figure 2). We resolved the members of the echinoid crown group with fairly high bootstrap support (73) and the clade of *Archaeocidaris* plus the crown group with even higher support (89). Our Bayesian analyses show similar results, with posterior probability (PP) of 0.85 for the clade of *Archaeocidaris* plus the crown group echinoids, and 0.6 for the clade of crown group taxa (figure 2). When five additional sensitivity analyses were run using varying values of the parameter *α* for the symmetric Dirichlet prior on character state transitions (electronic supplementary material, figure S2), we found a clade of *Archaeocidaris* and the crown group in all analyses. The PP for this clade was between 0.81 and 0.85 dependent upon which value of *α* was used. Furthermore, in 50% majority rule trees using all but one (*α *= 0.2) values of *α*, *Yunnanechinus* is resolved in the basal trichotomy, with a clade of all other taxa supported with PPs between 0.54 and 0.71.

## 5. Discussion

The end-Permian mass extinction, brought about by cataclysmic global climate change linked to Siberian Traps volcanic outgassing [24], represents the most significant culling of metazoan life in the Phanerozoic [25]. Though the extinction is widely accepted to have begun in the Changhsingian stage at the end of the Permian, the Early Triassic aftermath was characterized by repeated extinction events [26] and ecosystem recovery does not appear to have come to fruition until the Late Anisian, approximately 8 Myr after the onset of extinction [27]. The results of our phylogenetic analyses show that throughout this interval of global turmoil, a clade of stem group echinoids endured the onset of extinction during the latest Permian and persisted until at least the Middle Anisian. Furthermore, the Louping Biota was deposited in a low-energy, intracratonic basin [18], thus the risk of the fragile, imbricate-plated specimens of *Yunnanechinus* being reworked [17] is low to non-existent. Our phylogenetic analyses further re-affirm recent work [7] indicating that the extinction of stem group echinoids did not take place in the Palaeozoic, as has long been postulated. Thus, the long-utilized distinction between Palaeozoic and post-Palaeozoic echinoids is not analogous to the distinction between stem and crown group echinoids. Furthermore, our phylogenetic analyses consistently resolved a clade of *Archaeocidaris* and the crown group echinoids (figure 2, electronic supplementary material, figures S1–S3). The strong support for this clade indicates that the lineage which gave rise to *Yunnanechinus* must have diverged from the archaeocidarids and the crown group prior to the first occurrence of *Archaeocidaris* in the fossil record in the Tournaisian (Early Mississippian). Our sensitivity analyses using the alternative outgroup *Palaeodiscus ferox* (electronic supplementary material, figure S1) for parsimony analyses returned five MPTs. Therefore, although we are confident that *Yunnanechinus* is a stem group echinoid, its phylogenetic placement within the stem group remains tentative. We thus refrain from assigning it to a particular family, but note that it has plotted with both echinocystid and lepidocentrid echinoids (electronic supplementary material, figures S1–S3). *Yunnanechinus* was briefly mentioned previously [7] and referred to as a proterocidarid; however, our results show no support for the placement of this genus within the Proterocidaridae.

**Figure 3.**
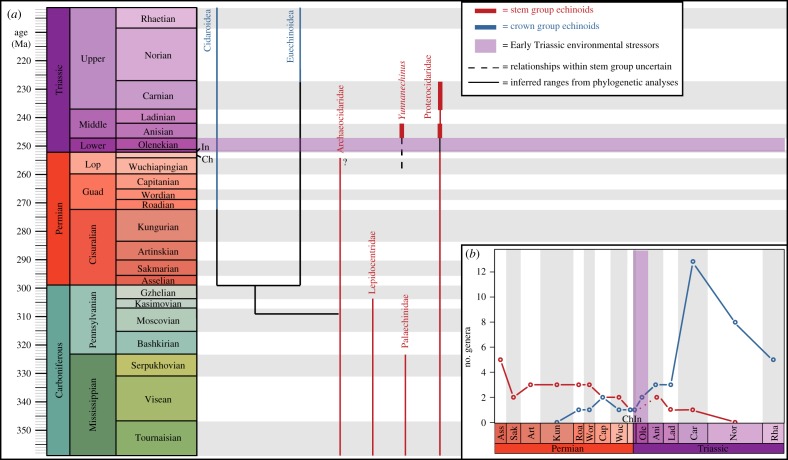
Diversity of echinoids from the Late Palaeozoic to the Early Mesozoic. (*a*) Stratigraphic ranges of echinoid taxonomic groups included in phylogenetic analyses. The phylogenetic relationship of *Yunnanechinus* to other stem group echinoids is uncertain, thus its phylogenetic relationships are depicted with a dashed line. The Early Triassic, which experienced significant environmental stress [4,27], lacks any fossil representation of stem group echinoids, which are Lazarus taxa during this time. (*b*) Diversity curve of stem group and crown group echinoids during the Permian and Triassic. The curve was compiled using the range through method [29]. The dashed line for stem group echinoids in the Induan and Olenekian represents the inferred presence of stem group precursors to *Yunnanechinus* but which have yet to be sampled from the fossil record. Crown group echinoids are first known from the Roadian, while stem group echinoids go extinct at the oldest in the Carnian. Throughout the entire figure, stem group echinoids are indicated in red and crown group echinoids in blue. Data are compiled from electronic supplementary material, tables S3 and S4, which list Permian and Triassic echinoid taxa.

Our analyses also show that stem group and crown group echinoids, which inhabited the same environments in the Permian [28], must have coexisted from the Roadian, when the first crown group echinoid is known from the fossil record, until at least the Anisian occurrence of *Y. luopingensis* (figure 3*a*). This implies an overlap in stratigraphic ranges of approximately 23 Myr between stem group and crown group echinoids (figure 3*b*), and potentially longer as suggested by disarticulated ossicles [7]. The presence of *Y. luopingensis* in the Middle Triassic now opens the door to questions regarding the diversity and distribution of stem group echinoids in the Mesozoic. With two Anisian occurrences, the stem group echinoids were more diverse and widely distributed in the Mesozoic than has hitherto been thought. This also raises the question: does the extinction of stem group echinoids have more to do with competitive replacement by crown group echinoids than with the end-Permian mass extinction? Although *Yunnanechinus* and the putative proterocidarid from the Muschelkalk [7] are the first stem group echinoids known from the Middle Triassic, numerous crown group echinoids have been reported from Anisian and Ladinian strata of the Muschelkalk Basin [30], Turkey [31] British Columbia [32] and China [33], all belonging to the subclass Cidaroidea. The diversity and abundance of cidaroids compared to these two stem group occurrences indicates that crown group echinoids attained much higher levels of diversity and a wider distribution than the stem group echinoids in the Triassic. Thus, despite having endured the end-Permian mass extinction, the stem group echinoids appear to have been ecologically minor members of Triassic ecosystems and these occurrences of stem group echinoids probably represent the last vestiges of a ‘dead clade walking’ [34], headed for extinction.

The occurrence of *Yunnanechinus* from the Anisian of South China also has novel implications for understanding the Permian–Triassic fossil record of echinoids. The youngest verified stem group echinoid known from the Palaeozoic is the Changhsingian proterocidarid *Pronechinus anatoliensi*s (figure 3*a*) [8]. This implies a minimum gap in the fossil record of approximately 7 Myr between the Anisian *Y. luopingensis*, the proterocidarid echinoid from the Muschelkalk, and the next oldest stem group echinoid in the fossil record. Stem group echinoids thus clearly exhibit the Lazarus effect [35] during the latest Permian and Early Triassic, having disappeared from the fossil record for the entire duration of the Early Triassic. This is in contrast to crown group echinoids, which have a Late Permian and Early Triassic fossil record of both articulated tests and disarticulated ossicles. Given that no stem group echinoids have been sampled from the fossil record of the Early Triassic and are now known again only from the Anisian supports the explanatory model of Wignall & Benton [36], where Lazarus taxa disappear from the fossil record during the interval of crisis, only to reappear during the phase of recovery. The Luoping Biota is thought to represent a recovered ecosystem [18,27], and that stem group echinoids have reappeared only when ecosystems appear to have stabilized is predicted by this model. Conversely, the occurrence of disarticulated echinoderm ossicles [7] and relatively complete crown group echinoid tests [16] in deeper water Triassic environments has led some authors to speculate that these habitats may have been refugia for stem group echinoids and other echinoderms during the Early Triassic. Whether or not the disappearance from the fossil record of stem group echinoids is due to mass rarity and low population size [36–38] or to the existence of habitable, but restricted, refugia in the Early Triassic [16,39] will remain the subject of further work. Given that stem group echinoids are now known from the Mesozoic, we urge palaeontologists and geologists working on Triassic strata to survey for stem group echinoids in the field to expand our collective knowledge of their diversity leading to their eventual extinction.

## Supplementary Material

Supplemental Table S1

## Supplementary Material

Supplemental Table S2

## Supplementary Material

Supplemental Table S3

## Supplementary Material

Supplemental Table S4

## Supplementary Material

Supplemental Nexus File 1

## Data accessibility

All data are available in the electronic supplementary material.
