# Supplementary material for: A new stem group echinoid from the Triassic of China leads to a revised macroevolutionary history of echinoids during the end-Permian mass extinction
Source: R Soc Open Sci. 2018 Jan 31;5(1):171548. doi: 10.1098/rsos.171548 (PMC5792935; doi:10.1098/rsos.171548)

A revised macroevolutionary history of echinoids during the end-Permian mass extinction Supplementary Material

Jeffrey R. Thompson[1], Shi-xue Hu[2,3], Qi-Yue Zhang[2,3], Elizabeth Petsios[1,4], Laura J. Cotton[4], Jin-Yuan Huang[2,3], Chang-yong Zhou[2,3], Wen Wen[2,3], David J. Bottjer[1]
[1]University of Southern California
[2]Chengdu Institute of Geology and Mineral Resources
[3]Chengdu Center of China Geological Survey
[4]University of Florida

Geological Setting Specimens of *Yunnanechinus luopingensis* n. sp. were collected from the Anisian Luoping biota of the Guanling Formation, which is known from eastern Yunnan and western Guizhou provinces. The Guanling formation is divided into two members, Member I and Member II and the Luoping biota is from the middle of Member II [1]. Member II has been constrained to the *Nicoraella kockeli* Zone based upon conodont biostratigraphy, which places its occurrence in the Pelsonian substage of the Anisian Stage of the Middle Triassic [2, 3]. The Luoping biota is a typical conservation Lagerstätte [4] with exceptionally preserved marine fossils and few terrestrial elements[1]. Well-preserved echinoderms are extremely rare, although disarticulated fragments of crinoids are common on particular bedding surfaces. So far only six echinoids have been recovered, all of which are individuals of *Y. luopingensis*. All specimens were recovered from two big quarries nearby the Dawazi Village, Luoping County, Yunnan Province.

Phylogenetic Analyses

Character Scoring

Because our phylogenetic analyses include both stem group and crown group echinoids, which have differing numbers of columns of both ambulacral and interambulacral plates, we needed to make decisions regarding the homology of these columns amongst stem group and crown group echinoids for character scoring. In our character scoring, we decided to treat the perradial columns of ambulacral plates in the stem group echinoids as homologous to those in the crown group taxa. Furthermore, adambulacral (adradial) most columns of interambulacral plates in each interambulacral are were treated as homologous amongst all included taxa. Additionally, although most characters in the phylogenetic analyses were treated as unordered, character number 59 was treated as ordered because interambulacral plates are added adapically adjacent to the ocular plates [5] and migrate adorally during growth. Any number of plates greater than one is the result of resorption [6], and is thus developmentally ordered. Nevertheless, parsimony analyses run with this character unordered returned the same topology.

Markov Chain Monte Carlo (MCMC)

MCMC was run for 12,000,000 generations, sampling every 500 generations. We used two chains, with four chains per run and the first 25% of samples were discarded as burn in. Convergence was assessed using the average deviation of split frequencies, as calculated by MrBayes, and chains were run until this was less than 0.01. Results of Bayesian analyses are presented as 50% majority rule trees, with clade credibility values (posterior probabilities) shown at resolved nodes.

Sensitivity Analyses

To assess the robustness of our results, we ran a number of sensitivity analyses to examine the effects of outgroup choice and model parameters on the phylogenetic position of *Yunnanechinus luopingensis* n. sp. as a stem group echinoid. The phylogenetic relationships of the major clades of stem-group echinoids are unclear, especially prior to the Carboniferous (Thompson pers observation.), thus we ran our analyses utilizing a different outgroup, the lepidocentrid echinoid *Palaeodiscus ferox.* When parsimony analyses in PAUP* were run with *Palaeodiscus ferox* as the outgroup, there were five resulting most parsimonious trees of length 116 steps with CI=0.707 and RI=0.752. These five trees and their strict consensus are shown in figure S1. Character matrices and lists used in analyses with both outgroups are shown in Tables S1 and S2.

We additionally ran additional Bayesian analyses utilizing different prior settings for analyses with both outgroups. The symmetric Dirichlet prior used in Bayesian analyses has one parameter, α, which determines the amount of asymmetry in character state transitions allowed for different characters in the dataset [7]. Larger values of α allow for more symmetry in transition rates while smaller values of α allow for more asymmetrical transition rates. For our sensitivity analyses, we ran five additional analyses for each outgroup using α values of 10, 2, 1, .2, and 0.05 following [7]. Majority rule trees resulting from these analyses are shown in figure S2 and S3.

Supplementary Figure Captions.

Figure S1. Most parsimonious trees resulting from sensitivity analyses using the lepidocentrid echinoid *Palaeodiscus ferox* as outgroup in parsimony analyses. (*a*) Strict consensus of the five most parsimonious trees with length=116 steps, consistency index (CI)=.707 and retention index (RI)=.752. Bootstrap proportions resulting from 10,000 “fast” bootstraps in PAUP*. Bootstrap proportions shown in bold, while decay indices (Bremer support) shown in italics. (*b*) One of the five most parsimonious trees. (*c*) One of the five most parsimonious trees. (*d*) One of the five most parsimonious trees. (*e*) One of the five most parsimonious trees. (*f*) One of the five most parsimonious trees.

Figure S2. Trees resulting from sensitivity analyses utilizing different values of the parameter α in the symmetric Dirichlet prior in Bayesian analyses with *Echinocystites ponum* used as the outgroup. All trees are 50% majority rule consensus trees with posterior probabilities shown at resolved nodes. Scales bars show branch lengths. (*a*) $\alpha=\infty$. (*b*) $\alpha=10$. (*c*) $\alpha=2$. (*d*) $\alpha=1$. (*e*) $\alpha=0.2$ (*f*) $\alpha=0.05$.

Figure S3. Trees resulting from sensitivity analyses utilizing different values of the parameter α in the symmetric Dirichlet prior in Bayesian analyses with *Palaeodiscus ferox* used as the outgroup. All trees are 50% majority rule consensus trees with posterior probabilities shown at resolved nodes. Scales bars show branch lengths. (*a*) $\alpha=\infty$. (*b*) $\alpha=10$. (*c*) $\alpha=2$. (*d*) $\alpha=1$. (*e*) $\alpha=0.2$ (*f*) $\alpha=0.05$.

Figure S4. Photos of type specimens of *Yunnanechinus luopingensis* n. sp. (*a*) Close up view of the apical and adapical test plating of the holotype, specimen LPI-32321. Scale is 1 mm. (*b*) Close up view of the apical area of LPI-32321 showing general plate with a single gonopore, the madreporite, and the imbricate plating of the adapical plates. Scale is 1 mm. (*c*) Test plating of

LPI-32321 showing the noncrenulate, imperforate tubercles, and the primary and secondary spines with their corresponding tubercles. Scale is 500 µm. (*d*) Spines and test plates of LPI-32321 showing the striate primary and secondary spines which lack milled rings. Scale is 500 µm. (*e*) view of specimen LPI-61166 showing a compressed test with spines. Scale is 1 cm. (*f*) Specimen LPI-61701, which is a counterpart of specimen LPI-61701 shown in Figure 1b. Scale is 1 cm.

References

[1] Hu, S.-x., Zhang, Q.-y., Chen, Z.-Q., Zhou, C.-y., Lü, T., Xie, T., Wen, W., Huang, J.-y. & Benton, M.J. 2010 The Luoping biota: exceptional preservation, and new evidence on the Triassic recovery from end-Permian mass extinction. *Proceedings of the Royal Society of London B: Biological Sciences*, rspb20102235.
[2] Zhang, Q.-y., Zhou, C., Lu, T., Xie, T., Lou, X., Liu, W., Sun, Y., Huang, J. & Zhao, L. 2009 A conodont-based Middle Triassic age assignment for the Luoping Biota of Yunnan, China. *Science in China Series D: Earth Sciences* **52**, 1673-1678.
[3] Zhang, Q.-y., Zhou, C., Lü, T., Xie, T., Lou, X., Liu, W., Sun, Y. & Jiang, X. 2008 Discovery and significance of the Middle Triassic Anisian biota from Luoping, Yunnan province. *Geological Review* **54**, 145-149. [In Chinese].
[4] Seilacher, A. & Westphal, F. 1971 "Fossil Lagerstätten". In *Sedimentology of parts of central Europe: International Sedimentological Congress VIII, Guidebook* (pp. 327-353. Frankfurt am Main, Verlag Waldemar Kramer.
[5] Zachos, L.G. & Sprinkle, J. 2011 Computational model of growth and development in Paleozoic echinoids. In *Computational Paleontology* (ed. A.M.T. Elewa), pp. 75-93, Springer.
[6] Jackson, R.T. 1912 *Phylogeny of the Echini: With a Revision of Palaeozoic Species*. Boston, The Boston Society of Natural History; 491 p.
[7] Wright, A.M., Lloyd, G.T. & Hillis, D.M. 2015 Modeling character change heterogeneity in phylogenetic analyses of morphology through the use of priors. *Systematic biology* **65**, 601-611.

Figure S1.

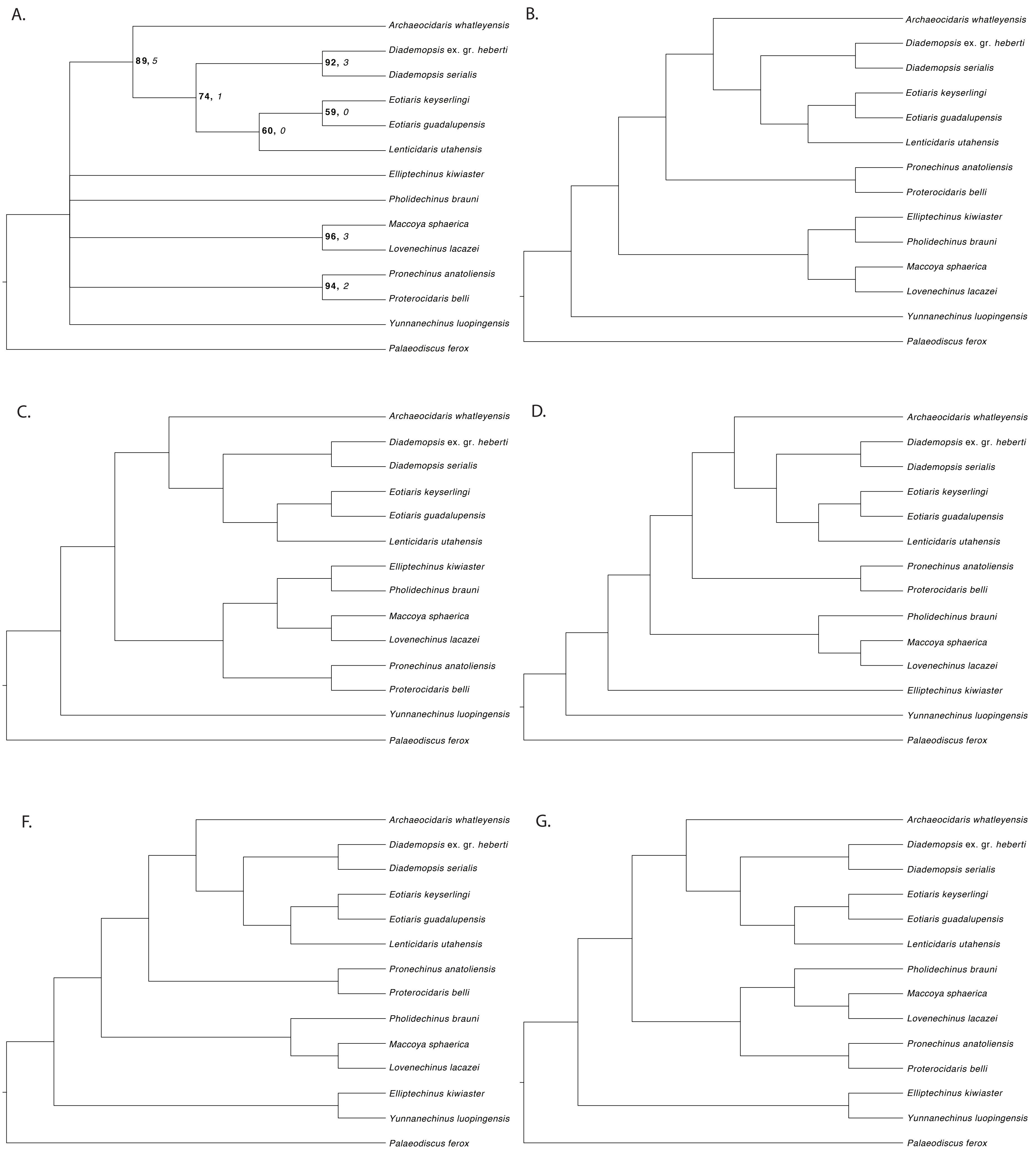
A.
Archaeocidaris whatleyensis
Diademopsis ex. gr. heberti
Diademopsis serialis
Eotiaris keyserlingi
Eotiaris guadalupensis
Lenticidaris utahensis
Elliptechinus kiwiaster
Pholidechinus brauni
Maccoya sphaerica
Lovenechinus lacazei
Pronechinus anatoliensis
Proterocidaris belli
Yunnanechinus luopingensis
Palaeodiscus ferox
89, 5
92, 3
74, 1
59, 0
60, 0
96, 3
94, 2
B.
Archaeocidaris whatleyensis
Diademopsis ex. gr. heberti
Diademopsis serialis
Eotiaris keyserlingi
Eotiaris guadalupensis
Lenticidaris utahensis
Pronechinus anatoliensis
Proterocidaris belli
Elliptechinus kiwiaster
Pholidechinus brauni
Maccoya sphaerica
Lovenechinus lacazei
Yunnanechinus luopingensis
Palaeodiscus ferox
C.
Archaeocidaris whatleyensis
Diademopsis ex. gr. heberti
Diademopsis serialis
Eotiaris keyserlingi
Eotiaris guadalupensis
Lenticidaris utahensis
Elliptechinus kiwiaster
Pholidechinus brauni
Maccoya sphaerica
Lovenechinus lacazei
Pronechinus anatoliensis
Proterocidaris belli
Yunnanechinus luopingensis
Palaeodiscus ferox
D.
Archaeocidaris whatleyensis
Diademopsis ex. gr. heberti
Diademopsis serialis
Eotiaris keyserlingi
Eotiaris guadalupensis
Lenticidaris utahensis
Pronechinus anatoliensis
Proterocidaris belli
Pholidechinus brauni
Maccoya sphaerica
Lovenechinus lacazei
Elliptechinus kiwiaster
Yunnanechinus luopingensis
Palaeodiscus ferox
F.
Archaeocidaris whatleyensis
Diademopsis ex. gr. heberti
Diademopsis serialis
Eotiaris keyserlingi
Eotiaris guadalupensis
Lenticidaris utahensis
Pronechinus anatoliensis
Proterocidaris belli
Pholidechinus brauni
Maccoya sphaerica
Lovenechinus lacazei
Elliptechinus kiwiaster
Yunnanechinus luopingensis
Palaeodiscus ferox
G.
Archaeocidaris whatleyensis
Diademopsis ex. gr. heberti
Diademopsis serialis
Eotiaris keyserlingi
Eotiaris guadalupensis
Lenticidaris utahensis
Pholidechinus brauni
Maccoya sphaerica
Lovenechinus lacazei
Pronechinus anatoliensis
Proterocidaris belli
Elliptechinus kiwiaster
Yunnanechinus luopingensis
Palaeodiscus ferox

Figure S2.

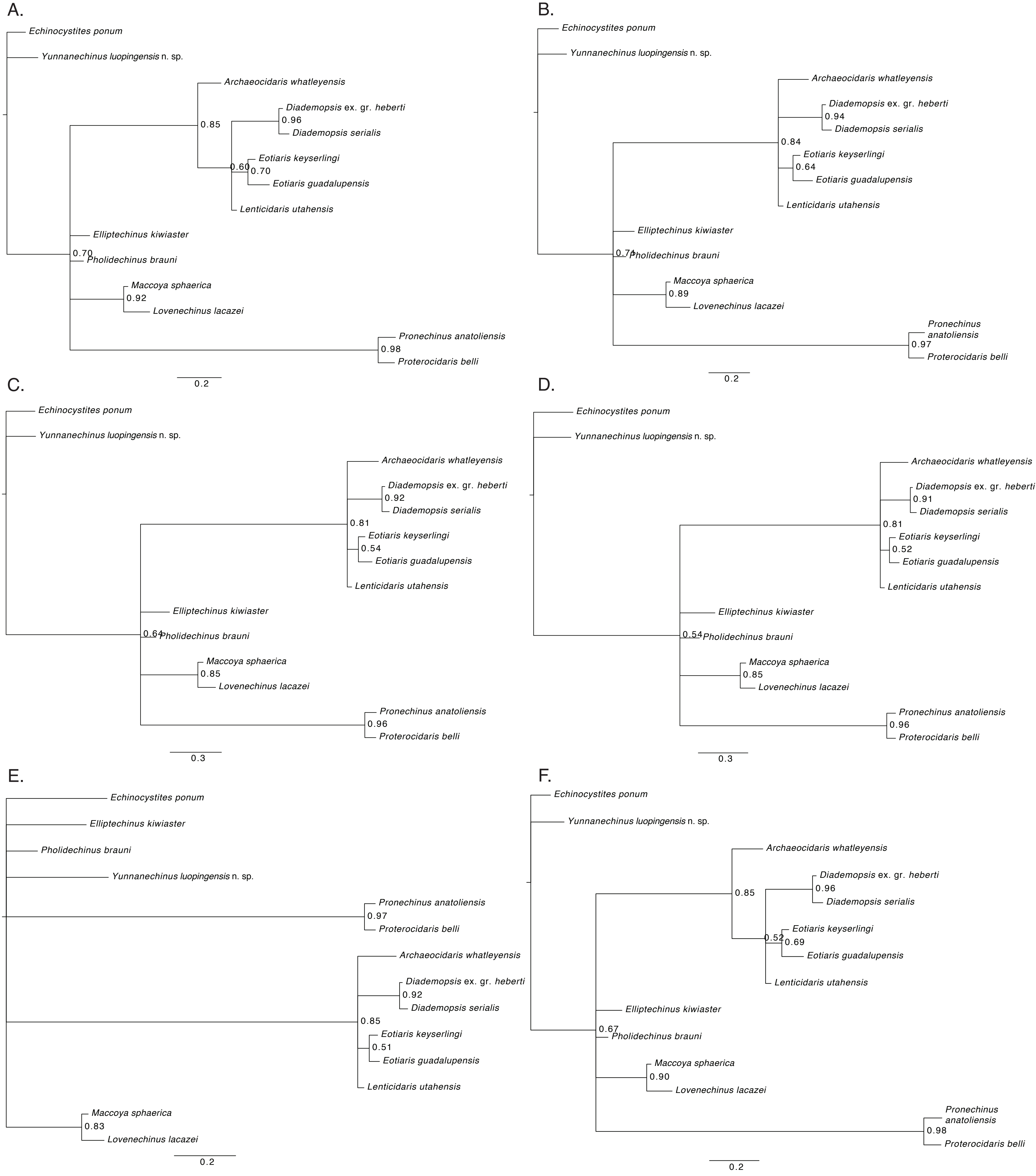
A.
Echinocystites ponum
Yunnanechinus luopingensis n. sp.
Archaeocidaris whatleyensis
0.85
Diademopsis ex. gr. heberti
0.96
Diademopsis serialis
Eotiaris keyserlingi
0.60
0.70
Eotiaris guadalupensis
Lenticidaris utahensis
Elliptechinus kiwiaster
0.70
Pholidechinus brauni
Maccoya sphaerica
0.92
Lovenechinus lacazei
Pronechinus anatoliensis
0.98
Proterocidaris belli
0.2
B.
Echinocystites ponum
Yunnanechinus luopingensis n. sp.
Archaeocidaris whatleyensis
Diademopsis ex. gr. heberti
0.94
Diademopsis serialis
0.84
Eotiaris keyserlingi
0.64
Eotiaris guadalupensis
Lenticidaris utahensis
Elliptechinus kiwiaster
0.74
Pholidechinus brauni
Maccoya sphaerica
0.89
Lovenechinus lacazei
Pronechinus anatoliensis
0.97
Proterocidaris belli
0.2
C.
Echinocystites ponum
Yunnanechinus luopingensis n. sp.
Archaeocidaris whatleyensis
Diademopsis ex. gr. heberti
0.92
Diademopsis serialis
0.81
Eotiaris keyserlingi
0.54
Eotiaris guadalupensis
Lenticidaris utahensis
Elliptechinus kiwiaster
0.64
Pholidechinus brauni
Maccoya sphaerica
0.85
Lovenechinus lacazei
Pronechinus anatoliensis
0.96
Proterocidaris belli
0.3
D.
Echinocystites ponum
Yunnanechinus luopingensis n. sp.
Archaeocidaris whatleyensis
Diademopsis ex. gr. heberti
0.91
Diademopsis serialis
0.81
Eotiaris keyserlingi
0.52
Eotiaris guadalupensis
Lenticidaris utahensis
Elliptechinus kiwiaster
0.54
Pholidechinus brauni
Maccoya sphaerica
0.85
Lovenechinus lacazei
Pronechinus anatoliensis
0.96
Proterocidaris belli
0.3
E.
Echinocystites ponum
Elliptechinus kiwiaster
Pholidechinus brauni
Yunnanechinus luopingensis n. sp.
Pronechinus anatoliensis
0.97
Proterocidaris belli
Archaeocidaris whatleyensis
Diademopsis ex. gr. heberti
0.92
Diademopsis serialis
0.85
Eotiaris keyserlingi
0.51
Eotiaris guadalupensis
Lenticidaris utahensis
Maccoya sphaerica
0.83
Lovenechinus lacazei
0.2
F.
Echinocystites ponum
Yunnanechinus luopingensis n. sp.
Archaeocidaris whatleyensis
0.85
Diademopsis ex. gr. heberti
0.96
Diademopsis serialis
Eotiaris keyserlingi
0.52
0.69
Eotiaris guadalupensis
Lenticidaris utahensis
Elliptechinus kiwiaster
0.67
Pholidechinus brauni
Maccoya sphaerica
0.90
Lovenechinus lacazei
Pronechinus anatoliensis
0.98
Proterocidaris belli
0.2

Figure S3.

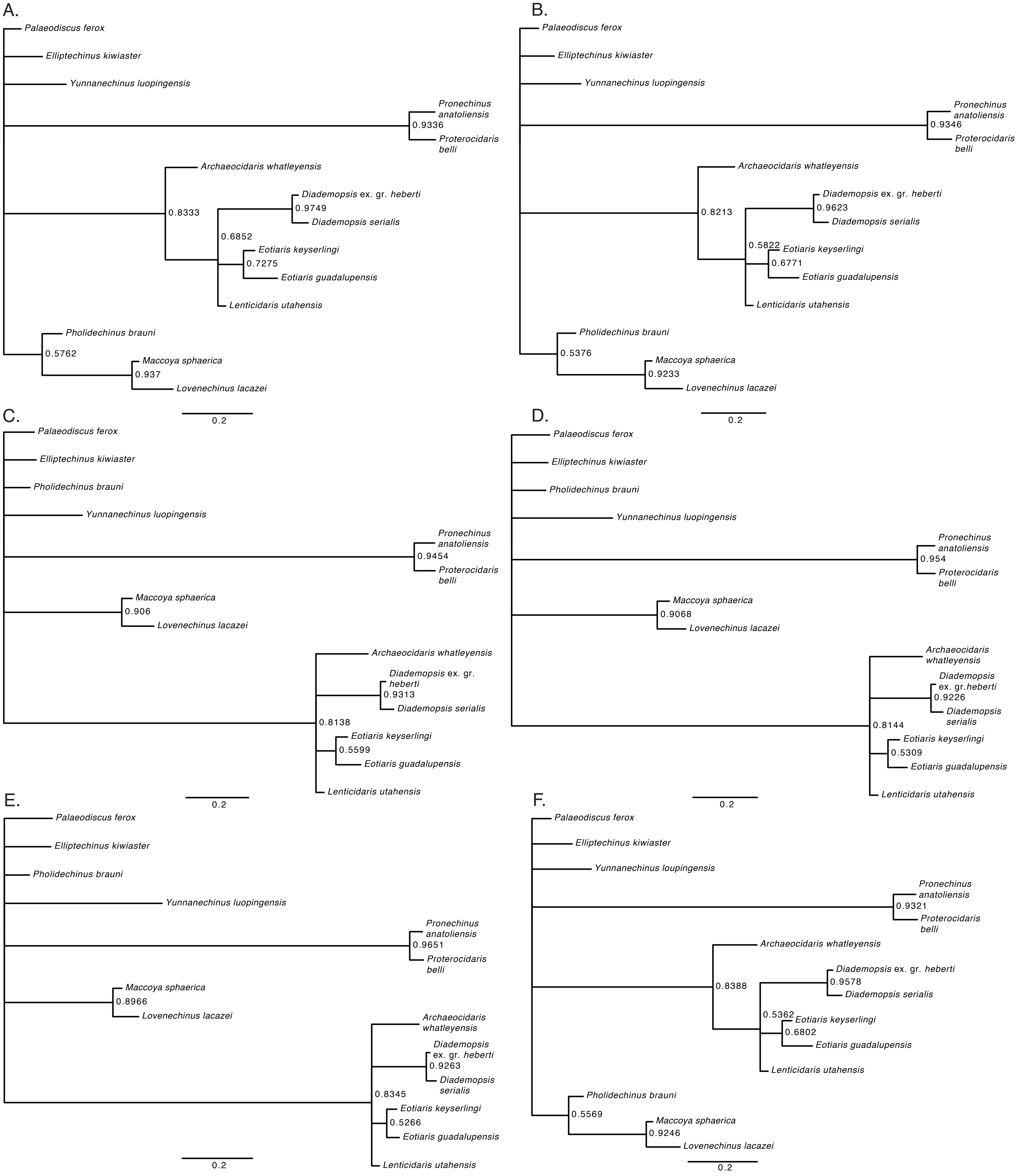
A.
Palaeodiscus ferox
Elliptechinus kiwiaster
Yunnanechinus luopingensis
Pronechinus anatoliensis
0.9336
Proterocidaris belli
Archaeocidaris whatleyensis
0.8333
Diademopsis ex. gr. heberti
0.9749
Diademopsis serialis
0.6852
Eotiaris keyserlingi
0.7275
Eotiaris guadalupensis
Lenticidaris utahensis
Pholidechinus brauni
0.5762
Maccoya sphaerica
0.937
Lovenechinus lacazei
0.2
B.
Palaeodiscus ferox
Elliptechinus kiwiaster
Yunnanechinus luopingensis
Pronechinus anatoliensis
0.9346
Proterocidaris belli
Archaeocidaris whatleyensis
0.8213
Diademopsis ex. gr. heberti
0.9623
Diademopsis serialis
0.5822
Eotiaris keyserlingi
0.6771
Eotiaris guadalupensis
Lenticidaris utahensis
Pholidechinus brauni
0.5376
Maccoya sphaerica
0.9233
Lovenechinus lacazei
0.2
C.
Palaeodiscus ferox
Elliptechinus kiwiaster
Pholidechinus brauni
Yunnanechinus luopingensis
Pronechinus anatoliensis
0.9454
Proterocidaris belli
Maccoya sphaerica
0.906
Lovenechinus lacazei
Archaeocidaris whatleyensis
Diademopsis ex. gr. heberti
0.9313
Diademopsis serialis
0.8138
Eotiaris keyserlingi
0.5599
Eotiaris guadalupensis
Lenticidaris utahensis
0.2
D.
Palaeodiscus ferox
Elliptechinus kiwiaster
Pholidechinus brauni
Yunnanechinus luopingensis
Pronechinus anatoliensis
0.954
Proterocidaris belli
Maccoya sphaerica
0.9068
Lovenechinus lacazei
Archaeocidaris whatleyensis
Diademopsis ex. gr.heberti
0.9226
Diademopsis serialis
0.8144
Eotiaris keyserlingi
0.5309
Eotiaris guadalupensis
Lenticidaris utahensis
0.2
E.
Palaeodiscus ferox
Elliptechinus kiwiaster
Pholidechinus brauni
Yunnanechinus luopingensis
Pronechinus anatoliensis
0.9651
Proterocidaris belli
Maccoya sphaerica
0.8966
Lovenechinus lacazei
Archaeocidaris whatleyensis
Diademopsis ex. gr. heberti
0.9263
Diademopsis serialis
0.8345
Eotiaris keyserlingi
0.5266
Eotiaris guadalupensis
Lenticidaris utahensis
0.2
F.
Palaeodiscus ferox
Elliptechinus kiwiaster
Yunnanechinus loupingensis
Pronechinus anatoliensis
0.9321
Proterocidaris belli
Archaeocidaris whatleyensis
0.8388
Diademopsis ex. gr. heberti
0.9578
Diademopsis serialis
0.5362
Eotiaris keyserlingi
0.6802
Eotiaris guadalupensis
Lenticidaris utahensis
Pholidechinus brauni
0.5569
Maccoya sphaerica
0.9246
Lovenechinus lacazei
0.2

Figure S4.

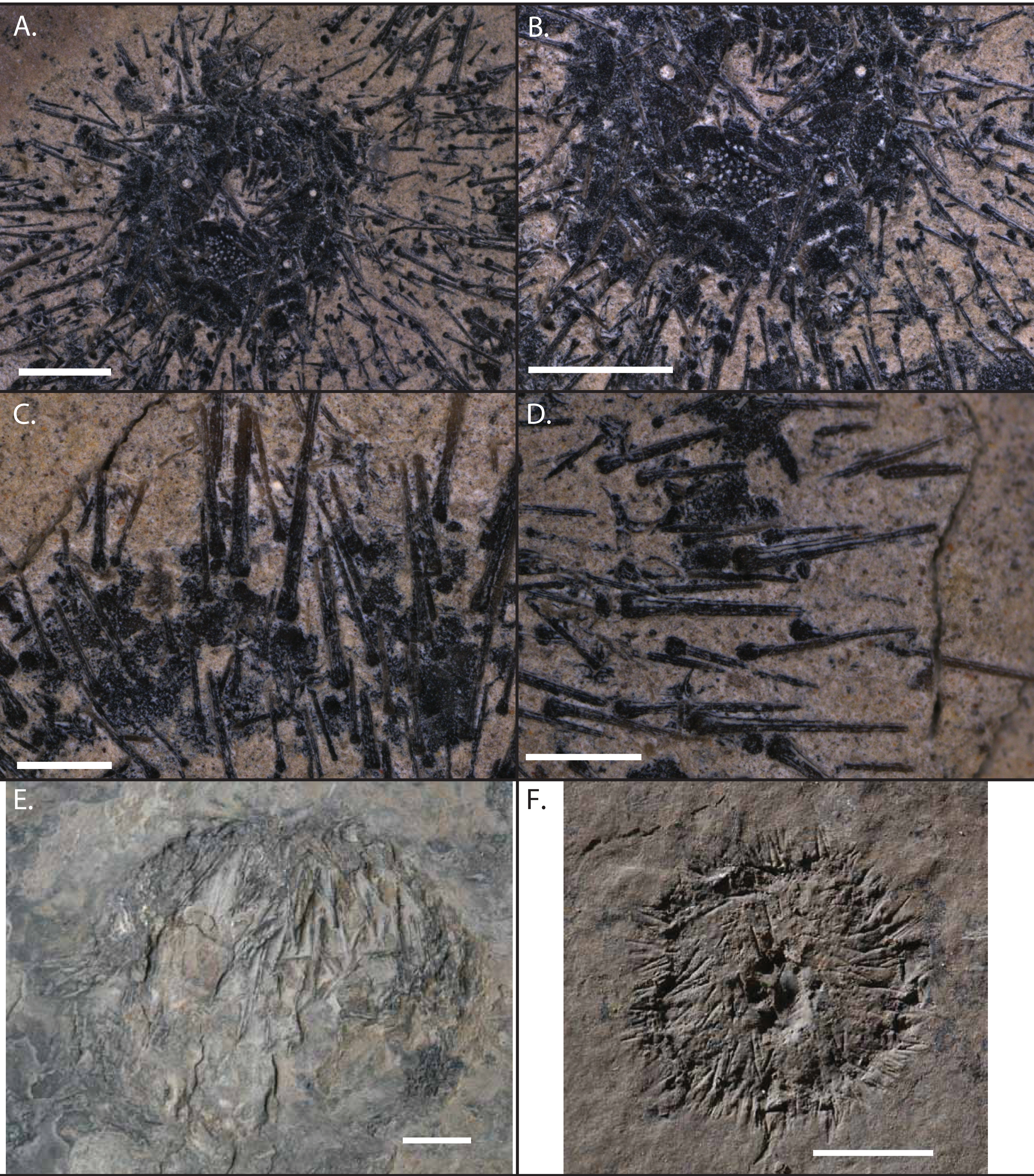

Supplement: Supplemental Nexus File 1 [file rsos171548supp5.pdf]
